# Editorial: Digital twins of plant and forest

**DOI:** 10.3389/fpls.2022.1049240

**Published:** 2022-12-23

**Authors:** Zhihan Lv, Houbing Herbert Song, Jun Shen, Neil Vaughan

**Affiliations:** ^1^ Department of Game Design, Faculty of Arts, Uppsala University, Uppsala, Sweden; ^2^ College of Engineering, Embry–Riddle Aeronautical University, Daytona Beach, FL, United States; ^3^ University of Wollongong, Wollongong, NSW, Australia; ^4^ University of Exeter, Exeter, United Kingdom

**Keywords:** digital twins, plantation management, precision control, data mining, GIS, data visualization

## 1 The background of this special section

Digital twins reflect the full life cycle of objects by mapping them into a virtual space. Forestry-related digital twins solutions provide easy access to 3D maps of forests or agriculture. This also presents enormous opportunities, from small features to macro tree inventory measurement capabilities. Additionally, experts can predict stress on large landscapes, generate ready-to-use weed control maps, and track changes to assets. In precision forestry and agriculture, digital twins can predict and cut business costs.

Building and dynamically updating a digital twins of a landscape in real time and optimizing woodland ownership from that digital twins becomes easier with digital twins technology solutions such as buffer analysis, landscape planning, inventory and forest harvesting planning, and timber inventory monitoring. From an environmental perspective, digital twins of forests play a vital role in understanding and monitoring the effects of drought and disease on trees. Insights from digital twins technology can reduce implant risk. In addition, the automated measurement of wood quantities also makes digital twins a very effective tool for assessing the carbon balance of forest lands.

## 2 The submission status of this special section

This special section attracted 10 submissions, in which 6 submissions are accepted.

## 3 An overview of each accepted papers

In “*Impact of Climate Change and Rubber (Hevea brasiliensis) Plantation Expansion on Reference Evapotranspiration in Xishuangbanna, Southwest China*”, the check.spatiotemporal variation of ET0 as well as its relationship in rubber plantations area in Xishuangbanna from 1970–2017 were analyzed by using trend, correlation and contribution analysis.

From the consideration of plants and forests, “*Applying Digital Twins to Research the Relationship Between Urban Expansion and Vegetation Coverage: A Case Study of Natural Preserve*”provides a comprehensive case study to research the relationship between urban development boundary and natural environment in a natural preserve in a coastal city.

In “*Population Structure and Spatial Distribution Pattern of Populus euphratica Riparian Forest Under Environmental Heterogeneity Along the Tarim River, Northwest China*”, terrestrial laser scanning (TLS) was applied to acquire a total of 1648 individual P. euphratica tree’s 3D structure attributes within 18 plots along the upper, middle, and lower reaches of the Tarim River.

“*Continuous Flat Pressing of MDF Quality Control Model Framework and Collaborative Programming Approach Based on Wood Fiber Hot Pressing Mechanism*” uses the continuous hot-pressing process of wooden medium-density fiberboard (MDF) to investigate the possibility of automatic quality control of the continuous flat pressing process.

“*A Case Study of the Relationship Between Vegetation Coverage and Urban Heat Island in a Coastal City by Applying Digital Twins*”has applied digital technologies to advance urban environmental research and forestry analysis.

In “*Forestry Digital Twin With Machine Learning in Landsat 7 Data*”, we propose a machine learning-based digital twin approach for forestry. A data processing algorithm was designed to process Landsat 7 remote sensing data as model’s input.

## Author contributions

ZL: Calling submission, handling review process, writing editorial HS: Calling submission, handling review process JS: Calling submission, handling review process NV: Calling submission, handling review process. All authors contributed to the article and approved the submitted version.

